# A configurable software platform for creating, reviewing and adjudicating annotation of unstructured text.

**DOI:** 10.23889/ijpds.v7i3.1953

**Published:** 2022-08-25

**Authors:** Richard Beare, Adam Morris, Tanya Ravipati, Elizabeth Le, Taya Collyer, Helene Roberts, Velandai Srikanth, Nadine Andrew

**Affiliations:** 1 Monash University; 2 NCHA; 3 Monash Health

## Objectives

To develop a flexible platform for creating, reviewing and adjudicating annotation of unstructured text. Natural Language Processing models and statistical classifiers use the results for analysis of large databases of text, such as electronic health records, that are curated by the National Centre for Healthy Ageing (NCHA) Data Platform.

## Approach

Automated approaches are essential for large scale extraction of structured data from unstructured documents. We applied the CogStack suite to annotate clinical text from hospital inpatient records based on the Unified Medical Language System (UMLS) for classifying dementia status. We trained a logistic regression classifier to determine dementia/non-dementia status within two cohorts based on frequency of occurrence of a set of terms provided by experts - one with confirmed dementia based on clinical assessment and the other confirmed non-dementia based on telephone cognitive interview. We used our annotation platform to review the accuracy of concepts assigned by CogStack.

## Results

There were 368 people with clinically confirmed dementia and 218 screen-negative for dementia. Of these, 259 with dementia and 195 without dementia had documents in the inpatient electronic health record system, 84045 inpatient documents 16950 for the dementia and non-dementia cohort respectively. A set of key words pertaining to dementia was generated by a specialist neurologist and a health information manager, and matched to UMLS concepts. The NCHA data platform holds a copy of the inpatient text records (>13million documents) that has been annotated using CogStack. Annotated documents corresponding to the study cohort were extracted.

We tested true positive rates of annotation against 50 concepts judged by a neurologist and health information manager to be relevant to dementia patients by manually review of 100 documents.

## Conclusion

Automated annotations must be validated. The platform we have developed allows efficient review and correction of annotations to allow models to be trained further or provide confidence that accuracy is sufficient for subsequent analysis. Implementation within our linked NCHA data platform will allow incorporation of text based data at scale.

